# Using Hologram-Based Augmented Reality in Anatomy Learning: The TEACHANATOMY Randomized Trial

**DOI:** 10.1097/ACM.0000000000006012

**Published:** 2025-03-03

**Authors:** Lukas Zingg, Melanie Häusler, Jonas Hein, Sascha Jecklin, Sören Kottner, Dominic Gascho, Nicola Cavalcanti, Phillippe Voinov, Tobias Götschi, Fabio Carrillo, Florian Lagler, Philipp Fürnstahl, Mazda Farshad

**Affiliations:** **L. Zingg** is assistant doctor, Balgrist University Hospital, University of Zurich, Zürich, Switzerland.; **M. Häusler** is research associate, Research in Orthopedic Computer Science, Medical Education, Balgrist University Hospital, University of Zurich, Zürich, Switzerland.; **J. Hein** is a PhD student, Research in Orthopedic Computer Science, Medical Education, Balgrist University Hospital, University of Zurich, Zürich, Switzerland.; **S. Jecklin** is a PhD student, Research in Orthopedic Computer Science, Medical Education, Balgrist University Hospital, University of Zurich, Zürich, Switzerland.; **S. Kottner** is an engineer, Institute of Forensic Medicine, University of Zurich, Zürich, Switzerland.; **D. Gascho** is technical manager of imaging and research group leader, Institute of Forensic Medicine, University of Zurich, Zürich, Switzerland.; **N. Cavalcanti** is clinical researcher, Research in Orthopedic Computer Science, Medical Education, Balgrist University Hospital, University of Zurich, Zürich, Switzerland.; **P. Voinov** is a software engineer, Research in Orthopedic Computer Science, Medical Education, Balgrist University Hospital, University of Zurich, Zürich, Switzerland.; **T. Götschi** is a statistician, Balgrist University Hospital, University of Zurich, Zürich, Switzerland.; **F. Carrillo** is research associate, Research in Orthopedic Computer Science, Medical Education, Balgrist University Hospital, University of Zurich, Zürich, Switzerland.; **F. Lagler** is professor, Department of Pediatrics and Institute for Inherited Metabolic Diseases, Paracelsus Medical University, Salzburg, Austria.; **P. Fürnstahl** is professor, Research in Orthopedic Computer Science, Medical Education, Balgrist University Hospital, University of Zurich, Zürich, Switzerland.; **M. Farshad** is medical hospital director and chief of orthopedics and spine surgery, University Hospital Balgrist, director, University Spine Center Zurich, and chair of orthopedics, University of Zurich, Zürich, Switzerland.

## Abstract

**Purpose:**

Augmented reality (AR) can generate realistic holograms overlaid on the real-word environment to provide an interactive learning experience. However, further research is needed to assess the impact of such technologies on knowledge acquisition. This study aims to evaluate the efficacy of an AR learning application in anatomy education.

**Method:**

TEACHANATOMY, a controlled, randomized trial, was conducted from September 14–October 24, 2022, at the University of Zurich and the Swiss Federal Institute of Technology. It included first- and second-year medical students with no prior anatomy education and compared learning with an interactive hologram-based AR learning module—incorporating adaptive learning and gamification elements—with traditional learning (TL) methods including textbooks, videos, and online resources. Forty-eight participants were randomly allocated to the AR or TL group. The primary outcome consisted of the scores on the theoretical and practical knowledge tests. Secondary outcomes included adverse health symptoms and user experience.

**Results:**

The AR group performed significantly better on both theoretical (median [IQR] scores: AR: 18.8 [16.6–20.0]; TL: 9.4 [7.7–11.3]; *P* < .001) and practical (AR: 14.0 [12.3–14.7]; TL: 5.0 [4.0–6.0]; *P* < .001) knowledge tests. The most common adverse health symptoms were headache, reported by 13/24 (54.2%) TL participants and 9/24 (37.5%) AR participants, and fatigue, and experienced by 13/24 (54.2%) TL participants and 2/24 (8.3%) AR participants. All participants evaluated learning with TEACHANATOMY as a positive experience, rating it as efficient and easy to understand. All participants agreed learning with AR can be beneficial in learning anatomy, with 89.6% (43/48) and 100% (48/48) expressing willingness to use AR as a learning tool for theoretical and practical anatomy learning, respectively.

**Conclusions:**

Findings show a short-term learning benefit with the use of the TEACHANATOMY learning application, thus supporting the implementation of interactive hologram-based AR technologies to improve knowledge in anatomy education.

Anatomy is central to medical training^1–3^ and is traditionally taught using lectures, textbooks, 3D models, prosections, and cadaver dissections. Cadaver dissections are considered the gold standard^4,5^ and are especially important to understand spatial relationships and individual variations of anatomical structures.^6^ However, there are several limitations to cadaver use in anatomy education. Cadaver labs are costly to maintain, require specialized supervision, and involve the use of harmful fixatives to preserve the bodies.^7–9^ In many countries, a shortage of bodies and ethical considerations represent an additional problem.^5,10,11^ Furthermore, dissections can be challenging and time-consuming for medical students and, if performed incorrectly, can prevent them from learning anatomical structures properly.^12,13^ Finally, many complex and important anatomical structures, like the nerves, are too small to observe in a cadaver.^7,12^

For these reasons, many universities have decreased the hours allocated to anatomy teaching in favor of applied clinical work,^14^ prompting the implementation of new learning methods based on visual technologies, such as augmented reality (AR).^14–20^ AR can generate realistic holograms that are overlaid in the real-word environment, merging the real and virtual worlds to provide an interactive learning experience.^21–23^ These technologies are already being tested in a wide range of medical areas and are becoming increasingly available as a teaching tool in medical education. Their use in education is still in its early stages, and although generally well received by students, further research is needed to assess their impact on knowledge acquisition.^16,21,23–28^

The TEACHANATOMY project aimed to develop an AR learning application to digitize cadavers and create an interactive hologram-based learning module focusing on the cranial nerves. To assess its efficacy in anatomy learning, we conducted a study comparing traditional learning (TL) methods, which included textbooks, videos, and online resources, with the novel AR learning module using the HoloLens 2 (Microsoft Corp, Redmond, Washington). We hypothesize that AR technology could improve anatomical knowledge and enhance student motivation and engagement by providing supplementary learning opportunities beyond the cadaver dissection lab.

## Method

This study was a prospective, controlled, randomized, 2-arm, parallel trial conducted at the Balgrist University Hospital from September 14 to October 24, 2022. Although involving 2 universities in Zurich, it was classified as a single-center trial due to the neuroanatomy course being a joint program between these institutions. The trial protocol is available as Supplemental Digital Appendix 1 (at http://links.lww.com/ACADMED/B685). The study followed the Consolidated Standards of Reporting Trials (CONSORT) extension guidelines (https://www.equator-network.org/reporting-guidelines/consort-routine/) and was approved by the Cantonal Ethics Committee of Canton Zurich (BASEC-Nr. Req-202200906).

### Participants

First- and second-year medical students from the University of Zurich and the Swiss Federal Institute of Technology with no prior anatomy education or experience with cadaver dissections were considered for enrollment. We recruited participants via flyers and WhatsApp messages 1 month before the intervention. Due to safety regulations for HoloLens 2 headsets, students with epilepsy, binocular vision disorder such as strabismus, current head and/or neck injuries, inflammation of the scalp and/or eye, and amputations or partial amputations of the hands were excluded. Participation was voluntary, and participants provided informed consent online without receiving any compensation.

### Randomization

We randomly allocated participants to either the TL (control) group or the AR group through an Internet-based concealed computer-generated random allocation sequence (Research Randomizer, Lancaster, Pennsylvania^29^).

### Training intervention

We conducted the study in 6 sessions spread over several days, each involving 8 participants, 4 assigned to the AR group and 4 to the TL group, making a total of 48 participants (24 in each group). All participants received a 10-minute general introduction to the study, followed by an introduction to their assigned study session (AR or TL; see Supplemental Digital Appendix 2 at http://links.lww.com/ACADMED/B685). For both groups, the study session was set up as an individual, self-directed session, with participants placed in separate rooms. To more closely simulate a library study setting, both groups had no time constraints during the study session and were allowed breaks. The learning module focused on the anatomy of the cranial nerves, with both groups receiving equivalent learning material in terms of the amount of information and level of detail presented. The equivalence of the learning material was assessed by the neuroanatomy course professor, an assistant professor, and a group of 3 students who had completed the neuroanatomy course. After reviewing the material, they agreed that the learning content was equivalent for both groups.

The study session for the TL group consisted of the most commonly used learning resources among students. To select these, we asked 80 nonparticipating students who had already completed the anatomy course which resources they found most helpful for learning neuroanatomy (see Supplemental Digital Appendix 3 at http://links.lww.com/ACADMED/B685). Based on the survey results, we provided the TL group participants with specific sections from 4 different neuroanatomy textbooks, access to 2 websites, 2 3D videos, and 2 online learning programs. All learning resources were available in both printed and PDF formats (all learning resources are summarized in Supplemental Digital Appendix 4 at http://links.lww.com/ACADMED/B685). Additionally, participants in the TL group were provided with a list of practice questions to check whether they had acquired the necessary information for the knowledge test.

**Figure 1:**
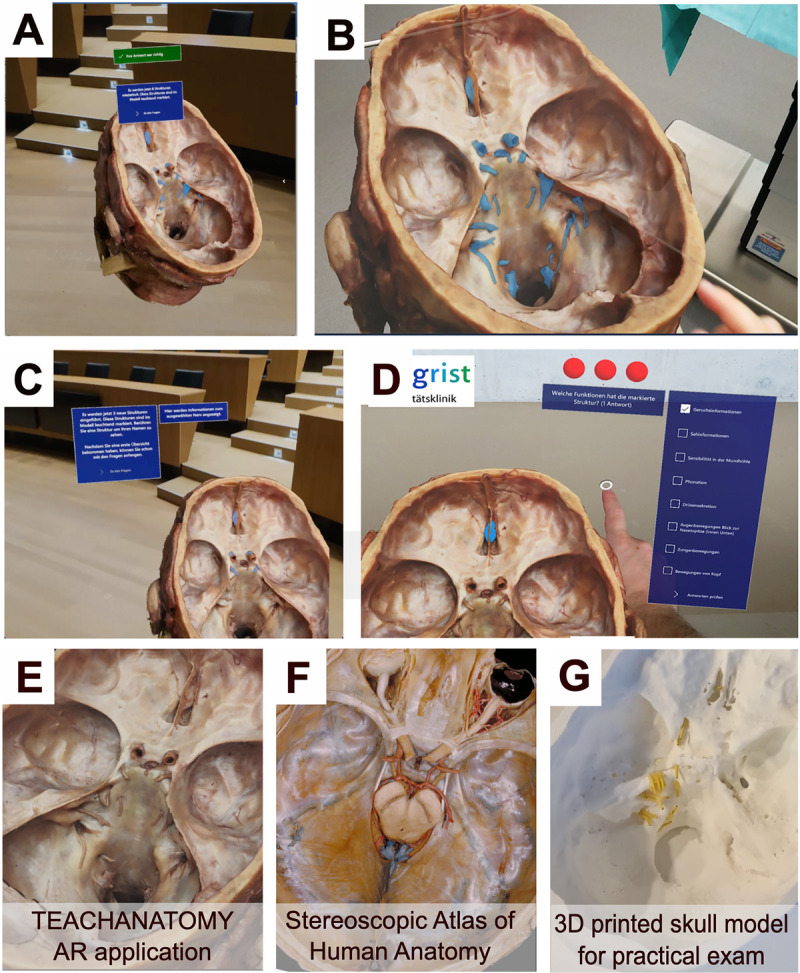
Representative images of the TEACHANATOMY learning application, randomized control trial comparing traditional learning methods with a novel augmented reality learning module, University of Zurich and the Swiss Federal Institute of Technology, September–October 2022. (Panels A–D) Representative images a participant would see while using the TEACHANATOMY learning application during the study session. Images of the skull as seen (panel E) in the TEACHANATOMY learning application, (panel F) in the “Basset Collection of Stereoscopic Images of Human Anatomy,” equivalent to the images used in the traditional learning group study session (image reproduced under the terms of the Creative Commons Attribution–ShareAlike 4.0 International License from: Stanford Medicine. Bassett Collection of Stereoscopic Images of Human Anatomy. Accessed February 19, 2025. https://lane.stanford.edu/biomed-resources/bassett/index.html); and (panel G) on the 3D printed anatomical skull model used for the practical section of the knowledge test.

In the AR group, participants were provided with a 20-minute tutorial to introduce the HoloLens 2 and the TEACHANATOMY learning application. The tutorial included initial steps to calibrate the headsets to ensure optimal functionality and comfort for each participant. Additionally, it provided comprehensive instructions on how to use the TEACHANATOMY application effectively. The study session consisted of 3 learning blocks, each lasting approximately 20 minutes, providing students with a comprehensive understanding of the anatomy of the cranial nerves, their function, and the typology, respectively. The learning blocks were followed by a repetition block of approximately 20 minutes, consisting of a series of questions to assess the acquired knowledge. In the learning blocks, the routes of the cranial nerves were projected onto the dissected head specimen. Users could click on these structures to learn their names, routes, and functions. The AR study session incorporated adaptive learning technology and gamification elements. Dialogue boxes prompted multiple-choice questions, and participants had 3 attempts to choose the correct answer. If they failed to select the correct answer within 3 attempts, they had to repeat the process. Hand control functionality allowed users to interact with holographic images, enabling actions such as resizing, rotating, and flipping plane axes. Additionally, participants could observe anatomical structures from different angles. To aid identification, the anatomical structures were highlighted and color coded (Figures 1A–1D and Supplemental Digital Appendix 5 at http://links.lww.com/ACADMED/B685).

For both the TL and AR groups, the study session was followed by a 30-minute knowledge test. Additionally, after the knowledge test, the TL group had an optional 30-minute demo session with the AR headsets featuring a shortened version of the learning module used for the AR group. The purpose of the demo session was to allow the TL group participants to experience the AR technology and showcase the features of the learning module.

At the end of the study, we provided all participants with a questionnaire to evaluate adverse health symptoms and user experience, followed by a 10-minute period for questions and feedback.

### Outcome measures

The primary outcome consisted of the scores on the final theoretical and practical sections of the knowledge test, assessed immediately after the study session. The questions on the theoretical and practical sections were developed together with the course professor and were representative of the actual exam questions currently used in the neuroanatomy course. The theoretical section consisted of 23 questions, requiring participants to recognize and name the 12 cranial nerves, differentiate their main functions and typology (sensory, motor, mixed), explain the relationships between their structure and function, and identify lesions using case studies. In the practical section (11 questions), participants were required to mark specific nerves on a 3D-printed anatomical skull model (Figures 1E–1G). The knowledge test questions are listed in Supplemental Digital Appendix 6A (at http://links.lww.com/ACADMED/B685), and the scoring system is detailed in Supplemental Digital Appendix 1 (at http://links.lww.com/ACADMED/B685). Briefly, for single-choice questions, correct answers scored 1 point, while wrong or unanswered questions scored 0. For multiple-choice questions, 1 point was awarded if correct answers equaled or exceeded wrong or unanswered responses; otherwise, 0 points were given.

Secondary outcomes included adverse health symptoms and user experience. Adverse health symptoms were evaluated through a survey assessing the presence and severity of symptoms rated on a Likert scale from 10 (almost imperceptible) to 100 (extreme; see Supplemental Digital Appendix 6B at http://links.lww.com/ACADMED/B685). User experience was assessed using an adapted NASA Task Load Index^30^ scale consisting of 6 questions with a score range of 1–10, where lower scores indicated lower cognitive workload. This scale is a widely used tool for evaluating perceived workload across various tasks and domains, including human-computer interaction and training scenarios like AR learning environments. Additionally, the user experience assessment included 10-point Likert scale items and open-ended questions in which participants were asked to rank their comfort with the material and hardware, as well as the learning effectiveness of the software in facilitating their understanding of neuroanatomy (see Supplemental Digital Appendix 6C at http://links.lww.com/ACADMED/B685).

### Creation of the 3D model

We created a 3D model using a fresh-frozen cadaveric head specimen donated for educational purposes within a university setting. We captured approximately 300 images using a digital single-lens reflex camera (Olympus OM-D E-M10), with a freehand, dome-like configuration surrounding the head to ensure full coverage from multiple angles. We then imported these images directly into RealityCapture software (version 1.4; RealityCapture EULA, Bratislava, Slovakia), which automatically generated the 3D model through photogrammetric processing (Figures 1A–1D).

### Software integration

The resulting 3D model was imported into Unity 3D (version 2021.3.8f1; Unity Technologies, San Francisco, California). Within Unity, anatomical regions, particularly the cranial nerves, were visually identified and segmented by applying specific textures to highlight each nerve. This allowed for clear visualization and interactive selection of individual cranial nerves, which was essential for educational purposes. An easy-to-use linking system was created within the application, enabling users to select individual nerves, providing access to detailed educational information about each nerve’s route and function (see Supplemental Digital Appendix 5 at http://links.lww.com/ACADMED/B685).

### Algorithmic framework

We integrated exam questions provided by the Institute of Anatomy, University of Zurich, into the AR learning application using an adaptive learning algorithm developed with the free, open-source application Anki (version 2.1.54; Anki Inc., San Francisco, California). This approach enabled automated tailoring of questions to match the user’s knowledge level. Users responded to each question by selecting the correct cranial nerve. The system then verified whether the user’s selection matched the correct texture annotation on the model and adjusted subsequent questions to align with the user’s demonstrated understanding.

### Output device

The Microsoft HoloLens 2 was chosen as the output device, enabling users to immerse themselves in a learning environment where they could physically move around the model to view it from different angles, akin to a real-life dissection. This interactive experience, where users actively selected and identified nerves, closely resembled traditional cadaveric dissections. After each learning session, metadata could be extracted via a USB-C connection for subsequent analysis. Although initially tailored for cranial nerves, this methodology can be adapted to other anatomical structures such as bones and muscles, making it a versatile tool for a wide range of educational applications.

### Sample size

Considering the normalized 0- to 10-point score ranges for the test outcomes, we estimated a minimally important difference of 0.6 points (SD = 7 points) between the final scores of the 2 randomized groups. Under these assumptions, a sample size of 24 participants per group provides 80% power (1 − *B* = 0.8) to detect a statistically significant difference between the groups at a significant level of .05 (alpha = .05), with an effect size of *d* = 0.83 (*t* test for 2 independent means).

### Statistical analysis

Prior to statistical analysis, we tested the data for normality with the Kolmogorov-Smirnov and the Shapiro-Wilk test for normality. We performed between-group comparisons using the Mann-Whitney test for continuous variables. Results were considered significant at a 2-tailed *P* < .05. We reported test scores as medians and interquartile ranges (IQRs) or numbers and percentages. We summarized adverse health symptoms both by the proportion of occurrence (in percentage) and by the median (IQR) severity of these occurrences. A cutoff point of 7 was defined for the user experience questionnaire scores. Scores of 7 or above indicated a positive experience, while scores below 7 were considered negative. The initial comparative analysis of participants’ characteristics was carried out with the chi-square or Fisher exact test. Effect size for the key outcomes was calculated with the Mann-Whitney *U* test, and internal consistency was assessed with Cronbach’s alpha. To determine the validity of the NASA Task Load Index, we calculated Spearman’s rho correlation. We conducted all analyses with SPSS Statistics (version 29; IBM, Armonk, New York), and we analyzed all data according to the intention-to-treat principle.

## Results

**Figure 2:**
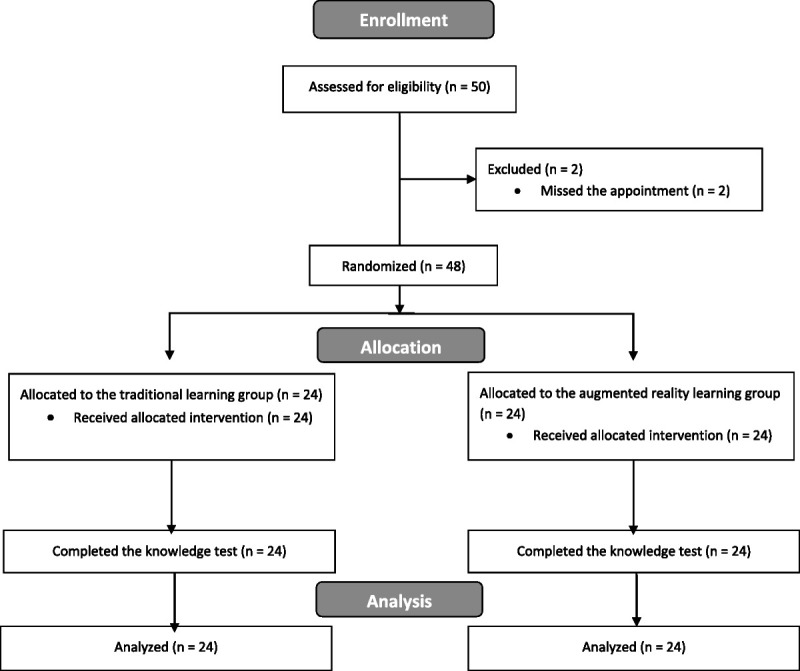
CONSORT 2010 (https://www.equator-network.org/reporting-guidelines/consort-routine/) participants flow diagram for a randomized control trial comparing traditional learning methods with a novel augmented reality learning module, University of Zurich and the Swiss Federal Institute of Technology, September–October 2022.

From September 14 to October 24, 2022, a total of 48 medical students participated in the study. The group included 21 (43.8%) men and 27 (56.3%) women (estimated mean age, 21.1 years^31^). The participant flow diagram is illustrated in Figure 2. We randomly allocated participants to either the TL group (24 participants: 11 [45.8%] women) or the AR group (24 participants: 16 [66.7%] women). Intergroup comparability showed that AR and TL groups were similar for all baseline characteristics except for the postgraduate training level, with the AR group having more first-year students than the TL group.

The neuroanatomy course is part of a joint program of the 2 Swiss universities in our study, being part of the first-year curriculum at one institution and the second-year curriculum at the other. Despite being in different academic years, all selected participants had no prior knowledge of neuroanatomy or experience with cadaver dissections and thus were considered equal in education level. Detailed demographic data are presented in Table 1.

**Table 1:** Demographic Characteristics of Study Participants^a^

Characteristic	TL participants (n = 24)	AR participants (n = 24)
**Estimated age (in years), mean^31^**	21.1	21.1
**Gender, no. (%)**		
Male	13 (54.2)	8 (33.3)
Female	11 (45.8)	16 (66.7)
**Postgraduate training level, no. (%)**		
First year	1 (4.2)	7 (29.2)
Second year	23 (95.8)	17 (70.8)
**Previously enrolled in another AR study, no. (%)**		
Yes	8 (33.3)	9 (37.5)
No	16 (66.7)	15 (62.5)
**Prior use of HoloLens 2 headset, no. (%)**		
Yes	1 (4.2)	0 (0)
No	23 (95.8)	24 (100)
**Previously enrolled in an anatomy course, no. (%)**		
Yes	0 (0)	0 (0)
No	24 (100)	24 (100)

Abbreviations: TL, traditional learning; AR, augmented reality.

^a^In a randomized control trial comparing TL methods with a novel AR learning module, University of Zurich and the Swiss Federal Institute of Technology, September–October 2022.

All participants included in the study completed the training (Figure 2). The overall duration of the study session, which included introductions, the study session, the knowledge tests, the voluntary demo session with AR for the TL group, the user experience and adverse health symptoms surveys, and the questions and feedback session, was between 3 and 4 hours.

### Knowledge test

**Figure 3:**
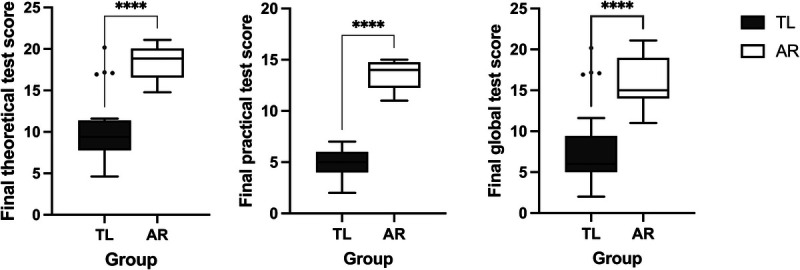
Final theoretical, practical, and global knowledge test scores of the TL and AR groups, randomized control trial comparing TL methods with a novel AR learning module, University of Zurich and the Swiss Federal Institute of Technology, September–October 2022. Lines within boxes indicate median values, lower and upper box edges are 25th and 75th percentile values, whiskers extend to ±1.5 times the interquartile range, and dots outside boxes are the most extreme values. Four asterisks indicate *P* < .001. Abbreviations: TL, traditional learning; AR, augmented reality.

Participants in the AR group achieved significantly higher final scores on both the theoretical (AR: median = 18.8 [IQR = 16.6–20.0]; TL: median = 9.4 [IQR = 7.7–11.3]; *P* < .001) and practical (AR: median = 14.0 [IQR = 12.3–14.7]; TL: median = 5.0 [IQR = 4.0–6.0]; *P* < .001) sections of the knowledge test compared to participants in the TL group (Figure 3). The internal consistency for the theoretical and practical sections was moderate (Cronbach’s alpha = .683) and strong (Cronbach’s alpha = .879), respectively. Results of the Mann-Whitney *U* test indicated that there was a significant difference between the 2 groups for the theoretical (*U* = 39.5, *z* = −5.125, *P* < .001), practical (*U* = 0.00, *z* = −5.9779, *P* < .001), and global (*U* = 2.00, *z* = −5.898, *P* < .001) scores with large effect sizes (see Supplemental Digital Appendix 7 at http://links.lww.com/ACADMED/B685).

### Adverse health symptoms

Overall, participants in the TL group reported experiencing more adverse health symptoms compared to those in the AR group (14 [58.3%] and 11 [45.8%] respectively; Table 2). The most common symptoms reported were headache, experienced by 13 (54.2%) participants in the TL group and 9 (37.5%) participants in the AR group, and fatigue, experienced by 13 (54.2%) participants in the TL group, but only 2 (8.3%) participants in the AR group. Most other symptoms also affected fewer participants in the AR group than in the TL group. For instance, 3 (12.5%) participants in the AR group reported neck stiffness/neck pain, compared with 9 (37.5%) in the TL group, and concentration problems were experienced by 1 (4.2%) participant in the AR group, compared to 4 (16.7%) in the TL group. Disorientation was only reported by 1 (4.2%) AR participant; no TL participants reported experiencing this symptom. None of the participants reported general discomfort during the study session. With respect to eye-related symptoms, 6 (25.0%) participants in the AR group reported blurred vision, compared to 1 (4.2%) in the TL group. One (4.2%) participant in the AR group experienced difficulty focusing, while 2 (8.3%) in the TL group reported this symptom. No participants in the AR group reported double vision or dry eyes, whereas in the TL group, 1 (4.2%) participant experienced double vision and 7 (29.2%) reported dry eyes. The internal consistency for the adverse health symptoms survey was weak (Cronbach’s alpha = .546).

**Table 2:** Adverse Health Symptoms Experienced by Study Participants^a^

Symptom	TL (n = 24)		AR (n = 24)	
	Participants, no. (%)	Severity, median [IQR]^b^	Participants, no. (%)	Severity, median [IQR]^b^
**General symptoms**				
General discomfort	0 (0)	—	0 (0)	—
Fatigue	13 (54.2)	56 [33]	2 (8.3)	41 [18]
Headache	13 (54.2)	33 [30]	9 (37.5)	41 [18]
Dizziness	2 (8.3)	10 [6]	1 (4.2)	18 [0]
Nausea	1 (4.2)	13 [0]	0 (0)	—
Concentration problems	4 (16.7)	49 [40]	1 (4.2)	58 [0]
Disorientation	0 (0)	—	1 (4.2)	17 [0]
Neck stiffness/neck pain	9 (37.5)	38 [32]	3 (12.5)	45 [40]
No symptoms	10 (41.7)	—	13 (54.2)	—
**Eye-related symptoms**				
Blurred vision	1 (4.2)	25 [0]	6 (25.0)	30 [22]
Difficulty focusing	2 (8.3)	47 [18]	1 (4.2)	27 [0]
Double vision	1 (4.2)	32 [0]	0 (0)	—
Dry eyes	7 (29.2)	59 [36]	0 (0)	—
No symptoms	15 (62.5)	—	17 (70.8)	—

Abbreviations: TL, traditional learning; AR, augmented reality; IQR, interquartile range.

^a^In a randomized control trial comparing TL methods with a novel AR learning module, University of Zurich and the Swiss Federal Institute of Technology, September–October 2022.

^b^Severity scale from 10 (almost imperceptible) to 100 (extreme).

### User experience

The results from the NASA Task Load Index revealed that learning with AR was associated with a significant decrease in overall workload score, with a median of 3.2 [IQR = 3.0–3.7] points in the AR group compared to a median of 4.4 [IQR = 3.8–5.0] points in the TL group (*P* < .001; see Supplemental Digital Appendixes 8 and 9 at http://links.lww.com/ACADMED/B685). The internal consistency for the NASA Task Load Index was good (Cronbach’s alpha = .763). To ensure the same directional interpretation for all questions, we reversed the scale of some questions before calculating the internal consistency. Results of the Mann-Whitney *U* test indicated significant differences between the 2 groups for most NASA Task Load Index questions, with large effect sizes indicating substantial differences in these variables. Exceptions included question 3 (*U* = 161.5, *z* = −2.634, *P* = .008), which had a medium effect size, suggesting moderate differences, and question number 4 (*U* = 256, *z* = −0.724, *P* = .469), which did not show a significant difference and had a small effect size, indicating little to no difference between the groups (see Supplemental Digital Appendix 10 at http://links.lww.com/ACADMED/B685). NASA Task Load Index test validation was calculated using Spearman’s rho correlation coefficient among the different questions. The significant correlations indicate that most of the NASA Task Load Index questions, with the exception of question number 3, were positively related to the total score (see Supplemental Digital Appendix 11 at http://links.lww.com/ACADMED/B685).

In comparison with the TL group, participants in the AR group rated the TEACHANATOMY learning experience as more emotional in any way (AR: median = 8.0 [IQR = 6.0–9.0]; TL: median = 4.0 [IQR = 3.2–5.0]; *P* < .001) and less mentally challenging (AR: median = 5.5 [IQR = 3.2–6.0]; TL: median = 7.5 [IQR = 7.0–8.0]; *P* < .001), and reported experiencing less frustration (AR: median = 3.0 [IQR = 1.2–5.0]; TL: median = 8.0 [IQR = 5.2–8.0]; *P* < .001). Both groups mostly rated the learning speed as “normal” (median = 5.0 [IQR = 5.0–6.0]). Participants in the AR group felt more confident in the subject area at the time of the survey (AR: median = 7.0 [IQR = 6.0–8.0]; TL: median = 4.5 [IQR = 3.0–6.0]; *P* < .001). When asked if they would take the opportunity to use AR to learn anatomy again in the future, the response was positive for 23 (95.8%) AR group participants (median = 9.75 [IQR = 8.5–10.0]). Overall, all participants evaluated learning with TEACHANATOMY as a positive experience, rating it as efficient and easy to understand. To the statement “I find it important to learn with the new technologies already employed in hospitals,” 43 of 48 (89.6%) gave a positive response. All participants agreed that learning with AR can be beneficial in learning anatomy, with 89.6% (43/48) and 100% (48/48) expressing willingness to use AR as a learning tool for theoretical and practical anatomy learning, respectively. All participants in the AR group felt they had achieved a substantial amount of learning. Similarly, participants in the TL group felt that learning with AR would have provided a more efficient and enjoyable learning experience, enabling them to master the subject more effectively than self-study (see Supplemental Digital Appendix 9 at http://links.lww.com/ACADMED/B685).

## Discussion

The TEACHANATOMY randomized trial assessed the effectiveness of an interactive hologram-based AR technology using the HoloLens 2 in anatomy learning. The study session focused on the neuroanatomy of the cranial nerves, regarded by medical students and early-career physicians as one of the most challenging portions of the anatomical sciences curriculum.^32^ Cranial nerves possess a high degree of anatomical and technical complexity, as well as significant anatomical variation. Moreover, due to their small size and complex pathways, cranial nerves are difficult to dissect. Therefore, new 3D technologies can be especially beneficial for mastering this subject.

Participants in the AR group achieved significantly higher final scores in both theoretical and practical knowledge test sections compared to those in the TL group (Figure 3), demonstrating a significant improvement in anatomical knowledge. Moreover, AR group participants experienced less adverse health symptoms than the TL group and found the learning experience significantly more enjoyable.

While recent studies and meta-analyses on the effectiveness of AR in medical education indicate a general improvement in motivation and engagement of participants,^16,17,33–41^ the learning benefits of these new technologies are still being debated, and many have found no significant learning advantages when using AR or virtual reality technologies instead of more traditional forms of learning.

Our results conflict with these findings, showing a clear learning benefit in the short term with the use of the TEACHANATOMY learning application, thus supporting its use to improve knowledge in anatomy education. This can be explained by the following reasons. First, traditional approaches rely heavily on passive learning through lectures, textbooks, and videos, which primarily offer 2D visualizations of anatomical structures. This makes the complexity of the spatial relationships between different structures harder to grasp, especially for individuals with limited spatial awareness.^42–45^ The TEACHANATOMY application, with its highly realistic 3D holograms, addresses this limitation, allowing students to visualize anatomical structures with the same definition and detail as in a dissection course. Users can move and rotate the holograms, and anatomical structures are colorized to aid identification, making them more user friendly than traditional models made from materials like wax, paper, and cardboard, or models that are 3D printed.

Second, the TEACHANATOMY application incorporated adaptive learning in both the study session and the final repetition block. In this approach, questions were modified based on participants’ performance. This allowed participants to spend less time with content they had already mastered and instead focus on improving in areas where they were weaker. Adaptive learning has been shown to increase confidence and at the same time decrease the cognitive load of participants.^46–49^ Third, the TEACHANATOMY application included gamification elements. Extensive research has shown that gamification has a positive impact on learning, effectively motivating and actively engaging students to perform better.^47,50,51^ Our use of these features in the TEACHANATOMY application may have greatly contributed to improving the AR participants’ knowledge and bolstering their confidence.

Unexpectedly, participants in the AR group experienced less adverse health symptoms than those in the TL group. However, since the adverse health symptoms survey showed weak internal consistency, the results derived from it should be interpreted with caution. Common adverse symptoms associated with the use of virtual reality and AR include headache, difficulty concentrating, dizziness, blurred vision, disorientation, and cybersickness.^52^ In our study, only 1 participant in the AR group reported experiencing disorientation, and the only other symptom more prevalent in the AR group than the TL group was blurred vision, reported by 25.0% of AR participants as opposed to 4.2% of TL participants. Blurred vision is possibly a consequence of the vergence-accommodation conflict, which occurs when the brain receives mismatching cues between the distance of a virtual 3D object and the focusing distance required for the eyes to focus on that object (i.e., users accommodate and converge to different distances).^53,54^ With the use of HoloLens 2, discomfort from the vergence-accommodation conflict can be minimized or avoided by placing holograms at a distance of at least 70 cm from the user, along with a careful eye/device calibration. This highlights the importance of performing a meticulous device calibration for each user to ensure a comfortable experience and prevent adverse health symptoms. Notably, fatigue was reported by 13 (54.2%) participants in the TL group, while 9 (37.5%) reported neck stiffness/neck pain, as opposed to 2 (8.3%) and 3 (12.5%) in the AR group, respectively. This difference may be attributed to the increased engagement and interactivity of the AR application, as well as the novelty effect. Moreover, participants in the TL group were mostly sitting during the study session, whereas the untethered AR headsets allowed AR study participants to move freely and interact hands-free with the holograms, thus decreasing the fatigue and neck stiffness/neck pain generally associated with prolonged sitting.^55–57^

Another important aspect highlighted by this trial is the positive user experience. Consistent with previous studies,^23,58–60^ participants enjoyed the experience and preferred this learning method over traditional approaches, rating it as efficient and less mentally challenging, with all participants in the AR group feeling they had achieved a substantial amount of learning.

The incorporation of AR technology in educational settings is particularly significant due to its novel approach to learning. AR offers an immersive and interactive experience that TL methods cannot replicate. By overlaying digital information onto the physical world, AR enables students to visualize and engage with complex concepts in a more intuitive and meaningful way. This approach has the potential to enhance understanding, retention, and engagement, providing a powerful tool for education. The introduction of new technologies, such as AR, into education is associated with initial high costs, with the average cost of a HoloLens 2 ranging from 4,000 to 5,000 euros (4,155 to 5,193 USD). However, despite this significant initial investment for the headsets, the learning material can be easily modified, updated, and extended at minimal additional costs, and with considerably less production time compared to traditional 3D models. Additionally, the AR content can be applied to all anatomical structures and programs, can be shared as an educational tool, and viewed remotely by multiple parties (e.g., during the COVID-19 pandemic), providing additional learning opportunities apart from the cadaver dissection lab. Furthermore, AR technologies have demonstrated great potential in improving spatial orientation and visualization, addressing the performance gap between students who struggle to visualize spatial structures and those confident in their spatial abilities.^42^ Finally, since visual technologies are already in use in medical training and in some surgical training procedures,^61–67^ incorporating AR within the medical curriculum might be beneficial to prepare students for a highly technologically enhanced workplace.

### Limitations

The results of this study should be interpreted with caution due to potential limitations. First, the study was considered a single site study with a single cohort and a small number of subjects, thus limiting the generalizability of our findings. Additionally, there may be significant variation in individual ability and performance on assessment tasks in general, as well as with AR technology. A second limitation was the absence of a follow-up test to assess the knowledge retention potential of AR-based learning. This is an important aspect that should be considered in future studies. Third, the repetition block in the AR study session included questions designed to assess and improve participants knowledge, implemented with adaptive learning technology and gamification elements. This could represent an advantage for AR group participants independent of the AR technology itself, introducing a potential bias. Finally, the novelty effect of the technology may have influenced participants’ satisfaction and should be taken into consideration, as it could introduce biases in the interpretation of user experience results.

### Conclusions

The TEACHANATOMY randomized trial suggests that the use of AR could significantly improve students’ knowledge of neuroanatomical structures. Moreover, it may increase participants’ confidence and engagement, while presenting only minor adverse health symptoms. Our findings support the implementation of interactive hologram-based AR technologies in health science and medical, and possibly other areas of, education.
